# Cognitive, Personality and Psychological Associations of Habit Formation and Change in Ageing

**DOI:** 10.1093/geroni/igaf122.2168

**Published:** 2025-12-31

**Authors:** Anangsha Pathak, Daniel Hermens, Daniel Fassnacht, Thomas Pace, Sanne de Wit, Sophie Andrews

**Affiliations:** University of the Sunshine Coast Thompson Institute, Birtinya, Queensland, Australia; University of the Sunshine Coast Thompson Institute, Birtinya, Queensland, Australia; University of the Sunshine Coast, Sunshine Coast, Queensland, Australia; University of the Sunshine Coast Thompson Institute, Birtinya, Queensland, Australia; University of Amsterdam, Amsterdam, Noord-Holland, Netherlands; University of the Sunshine Coast Thompson Institute, Birtinya, Queensland, Australia

## Abstract

All behaviour can be influenced by automatic, context-dependent habits. The ability to form and modify these habits is crucial for healthy ageing. Whilst cognition and personality can impact habit-related behaviours, their specific roles remain unclear. This study aimed to explore the relationships between cognitive, psychological, and personality factors in habit formation and change among older adults. A total of 125 healthy adults (Mage = 72.8, range = 60–87 years, SD = 7.25, 50.4% female) participated. Participants completed a computerised task comprising a habit learning phase and a habit switching phase (Symmetrical Outcome Revaluation Task), along with assessments of personality (IPIP-NEO), cognitive function (HVLT, Trails B), mood (DASS-21), and single-item scales for motivation, enjoyment, and satisfaction. Backward stepwise structural equation modelling was conducted and yielded an overall good model fit (indicated by, for example, the Comparative Fit Index =.956, reflecting the proportion of variance explained compared to a baseline model, with values above 0.90 indicating a good fit). The analysis revealed that cognitive and psychological factors significantly influenced habit learning and behavioural flexibility. Specifically, lower age, faster processing speed, better execute function, more enjoyment, motivation and lower depression was associated with improved habit learning accuracy (R² = .3). Additionally, better verbal recognition memory, higher conscientiousness, apathy and faster discrimination accuracy were linked to better performance in switching from habitual to goal-directed responding (R² = .11). These findings suggest that a combination of cognitive and psychological factors influence habit learning and behaviour change, with important implications for promoting healthy habits in ageing.

